# Clinical and Patient‐Reported Outcome Measures of Palatal Donor Site Healing Using Polyvinylpyrrolidone–Sodium Hyaluronate Gel as a Dressing Material Following Free Gingival Graft Harvesting: A Randomized Controlled Clinical Trial

**DOI:** 10.1002/cre2.70026

**Published:** 2024-11-11

**Authors:** Mohammad Baroudi, Majd Othman

**Affiliations:** ^1^ Department of Periodontology, Faculty of Dental Medicine Damascus University Damascus Syria

**Keywords:** pain, palate, wound healing

## Abstract

**Objectives:**

This study evaluates the efficacy of polyvinylpyrrolidone–sodium hyaluronate (PVP‐SH) gel as a dressing material in enhancing both clinical and patient‐reported outcomes post‐free gingival graft (FGG) harvesting from the hard palate.

**Material and Methods:**

This randomized clinical trial included 18 patients and spanned from June 2022 to August 2023. Patients received an FGG procedure to manage a lack of attached gingiva. Following graft harvesting, patients were divided into two groups: the control group, which received Coe‐Pak, and the test group, which was treated with PVP‐SH gel. The primary outcome measured was complete epithelialization. Secondary outcomes included the Landry healing scale, post‐operative pain level, and patient willingness to receive the same treatment again.

**Results:**

The test group reported significantly lower analgesic use (*p* < 0.001) and reduced pain severity (*p* < 0.001) compared to the control group. Furthermore, test group patients indicated a higher level of satisfaction regarding the prospect of retreatment. In contrast, the control group showed significantly slower progress in wound healing and epithelization (*p* < 0.05) compared to the test group.

**Conclusions:**

The findings of this study suggest that PVP‐SH gel is a superior dressing material post‐FGG harvesting, yielding improved clinical and patient‐reported outcomes relative to Coe‐Pak.

## 1 Introduction

One of the foundation treatment choices of periodontal plastic surgery is the utilization of free gingival grafts (FGGs) to reconstruct damaged periodontal structures (Meza‐Mauricio et al. 2021). The hard palate is often chosen as the harvest site for FGGs due to the superior characteristics of its keratinized gingiva (Parvini et al. 2021). However, this procedure is associated with several drawbacks, including patient discomfort, post‐surgical bleeding, and exposure of a second surgical site to the oral environment (Keceli et al. 2015). The donor site's healing time is estimated at 2–4 weeks when left to secondary intention healing (Del Pizzo et al. 2002), and patients frequently report significant pain and post‐operative bleeding (Tawfik 2020). These challenges have prompted the scientific community to seek alternatives to FGGs that eliminate the need for palatal harvesting and reduce patient discomfort. Despite considerable efforts, no current market material matches FGGs' outcomes. Recent advancements include the use of acellular dermal matrix grafts, collagen matrices, and polymeric matrices of human amniotic membranes, which have shown promising results, but still do not fully replicate the results achieved with FGGs (Dragan et al. 2017; Rotundo et al. 2024).

An alternative approach has been to develop a suitable barrier material to cover the donor site, protecting it from the oral environment and mitigating pain and discomfort. Many studies have documented the utilization of various materials as barriers for the donor site, including Cyanoacrylate (Veríssimo et al. 2021), collagen plugs (Basma et al. 2023), blood concentrates such as platelet‐rich fibrin (PRF; Shakir et al. 2015) membranes and leukocyte‐rich PRF (L‐PRF; Gatti et al. 2023), absorbable collagen wound dressings (e.g., HeliCOTE), and oxidized regenerated cellulose (e.g., Surgicel) (Silva, de Souza, and Nogueira 2023). These products have entered the market aiming to achieve this goal.

Polyvinylpyrrolidone–sodium hyaluronate (PVP‐SH) gel is a dense oral gel designed to form a protective barrier for alleviating pain by forming a protective layer on the oral mucosa (Buchsel, 2008). The mucosal barrier gel consists of three primary components: polyvinylpyrrolidone (PVP), sodium hyaluronate, and glycyrrhetinic acid. PVP is a hydrophilic and hygroscopic polymer known for its chemical and biological neutrality. Its wetting characteristics enable the gel to adhere to mucosal surfaces and form a protective film, which may improve tissue moisture retention. This polymer is also used as a binding agent in tablet formulations due to its inert properties. Sodium hyaluronate, or hyaluronic acid, is a viscous polysaccharide found naturally in various body tissues including the skin, ocular fluid, and joint lubricant. It functions as a film‐forming, mucoadhesive substance that physically shields the oral mucosa. In conditions mimicking physiological pH and ionic concentration, sodium hyaluronate transforms into a viscoelastic solution that may act as a lubricant and further promote tissue hydration. Glycyrrhetinic acid, a hydrophobic pentacyclic triterpenoid derived from glycyrrhizin, shares structural similarities with corticosteroids (Buchsel, 2008).

Several clinical studies have assessed the efficacy of PVP‐SH gel in mitigating pain from oral mucositis (OM), mouth ulcers, and wounds, including aphthous ulcers. In a small‐scale prospective trial with 30 participants, DeCordi and Martina observed patients undergoing chemotherapy for various cancers, such as rectal, lung, stomach, and head and neck cancers. They reported that within 3 days of applying the gel, there was a notable decrease in OM severity (57%), pain reduction (83%), and enhanced ability to consume food and beverages (83%) (De Cordi et al. 2001). Another study by Innocenti, Moscatelli, and Lopez involved 30 individuals with painful mouth and oropharyngeal inflammatory and ulcerative conditions treated with the gel. They utilized a visual numeric scale from 1 to 10 to gauge OM pain intensity, with 10 indicating extreme pain. Among these patients, six were suffering from OM due to cancer treatments. A significant drop in average pain scores by 92% was recorded within 5–7 h after the initial application of the gel. All participants noted a marked pain relief, with 40% experiencing optimal effects lasting 2–3 h and 57% feeling relief for over 3 h. After a week of treatment, an improvement in baseline pain scores and swallowing discomfort was reported by 87% of the patients (Innocenti, Moscatelli, and Lopez 2002).

Although the material in question is not novel, its clinical efficacy for alleviating pain and promoting healing at the donor site of FGGs remains underexplored, with limited research available. Our study endeavors to fill this knowledge gap by rigorously evaluating the use of PVP‐SH gel as the test dressing in managing the donor site post‐FGG harvest. The control dressing used was Coe‐Pak, a commonly used periodontal dressing. Specifically, we aimed to assess the complete epithelialization (CE), Landry healing scale, post‐operative pain level, and patient willingness to receive the same treatment again. The null hypothesis was that there would be no significant difference in the clinical and patient‐reported outcomes between the test and control groups.

## 2 Materials and Methods

### 2.1 Study Design and Sample Size Calculation

We conducted a randomized controlled trial (RCT) with a parallel arm design to assess specific outcomes: CE, Landry healing scale, post‐operative pain level, and patient willingness to receive the same treatment again, which are associated with two different wound dressings applied at the palatal donor site after FGG harvesting. The two study groups were as follows:

1. **Coe‐Pak (Control Group):** Participants in this group received the standard dressing.
2. **PVP‐SH gel (Test Group):** Participants in this group received the experimental dressing.

This study was carried out at the Department of Periodontology, Faculty of Dental Medicine, Damascus University, Syria, from June 2022 to August 2023. All participants received comprehensive information about the procedures, potential side effects, risks, benefits, and study timeline. Before the first phase of the study, each participant provided signed informed consent. The study protocol was reviewed and approved by the Ethics Committee of Damascus University (UDDS‐28066141/SRC‐2215). Our reporting adhered to the checklist items proposed in the CONSORT statement. The sample size was calculated using G*Power (version 3.1.9.7, Germany). Based on a previous study by Raghavan et al. (2020), an effect size of 1.564 was determined. A sample size of 12 patients (6 per group) was deemed sufficient to achieve a Type I error rate of 5% and a power of 80%. To account for a potential 20% dropout rate, the sample size was increased to 18 patients (9 per group). In this study, we did not include a third group with no treatment applied to the donor site due to ethical considerations. Leaving the donor site untreated could result in unnecessary pain and discomfort for the patients, which would not be in line with ethical research practices. Additionally, previous studies have already established the baseline healing process for untreated donor sites (Silva, de Souza, and Nogueira 2023), allowing us to focus on comparing the efficacy of the two treatment modalities (PVP‐SH gel and Coe‐Pak) without compromising patient well‐being.

This randomized clinical trial (RCT) employed a simple randomization method to allocate patients into the control and test groups. A computer‐generated randomization sequence was used to ensure unbiased allocation. Allocation concealment was maintained using sealed, opaque envelopes containing the group assignments. These envelopes were prepared by an independent researcher who was not involved in this study. The envelopes were opened sequentially by the operator at the time of the intervention, ensuring that the allocation remained concealed until the point of treatment. This approach minimized selection bias and ensured the integrity of the randomization process.

### 2.2 Eligibility Criteria

Patients were eligible to participate in the study based on the following criteria:

1. Age: Participants must be at least 18 years old.
2. Systemic health: Patients without any systemic diseases were considered eligible.
3. Palatal tissue thickness: The palatal soft tissue thickness should be at least 2 mm, assessed by bone sounding using a periodontal probe.

Patients were excluded from the study if they met any of the following criteria:

1. Smoking: Individuals who smoke.
2. Coagulation disorders: Patients who are unable to undergo surgical procedures due to coagulation disorders.
3. Anticoagulant therapy: Patients who are currently receiving anticoagulant therapy.
4. Compromised healing ability: Individuals with compromised healing ability, such as Type II diabetes mellitus.

### 2.3 Surgical Procedures, Donor Site Management, and Post‐Surgical Interventions

**Figure 1:**
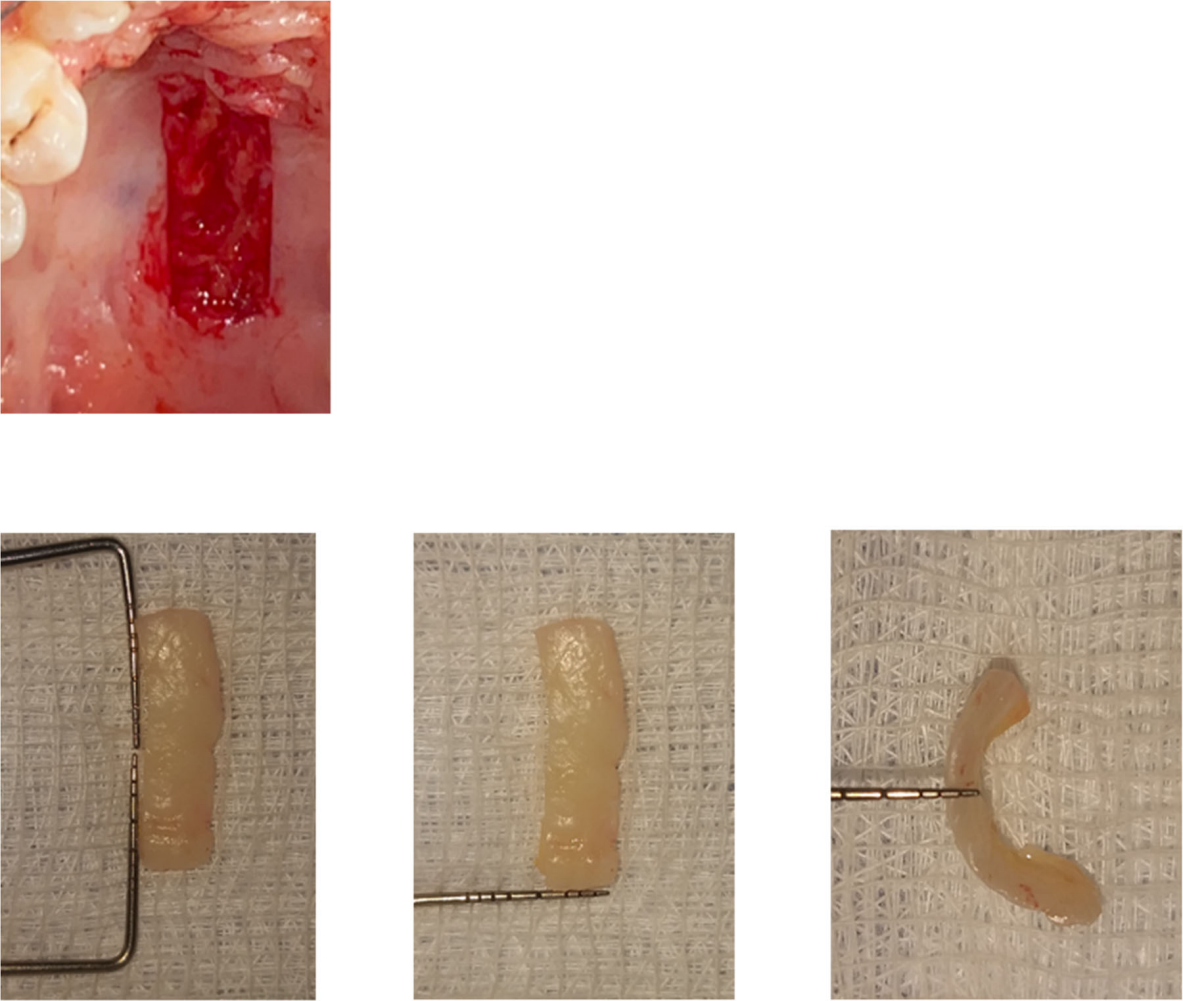
The harvesting and measurements of length, width, and thickness of the graft.

All eligible patients received the same surgical intervention: an FGG harvested from the hard palate. The purpose of this intervention was to manage a lack of attached gingiva on a single tooth in the anterior region of the mandible. Before the surgical procedure, patients were instructed to use a 2% povidone‐iodine mouthwash for 1 min. The surgical procedures were performed under local anesthesia using 2% lidocaine (1:80,000; Kwang Myung Pharm, Sindaebang 1‐dong, Dongjak‐gu, Seoul, Korea). The recipient sites were prepared following the technique described by Langer and Langer (1985). For both groups, FGGs were harvested following the technique described by Sullivan and Atkins (1968) (Figure 1). The grafts were standardized to approximately 2 mm in thickness using an endodontic file and a caliper. Measurements of length, width, and thickness were recorded for each graft (Figure 1). Finally, the grafts were transported to the recipient sites, adjusted as needed, and sutured in place using resorbable sutures. All surgical interventions and measurements in this study were performed by the same operator to ensure consistency and reduce inter‐operator variability.

**Figure 2:**
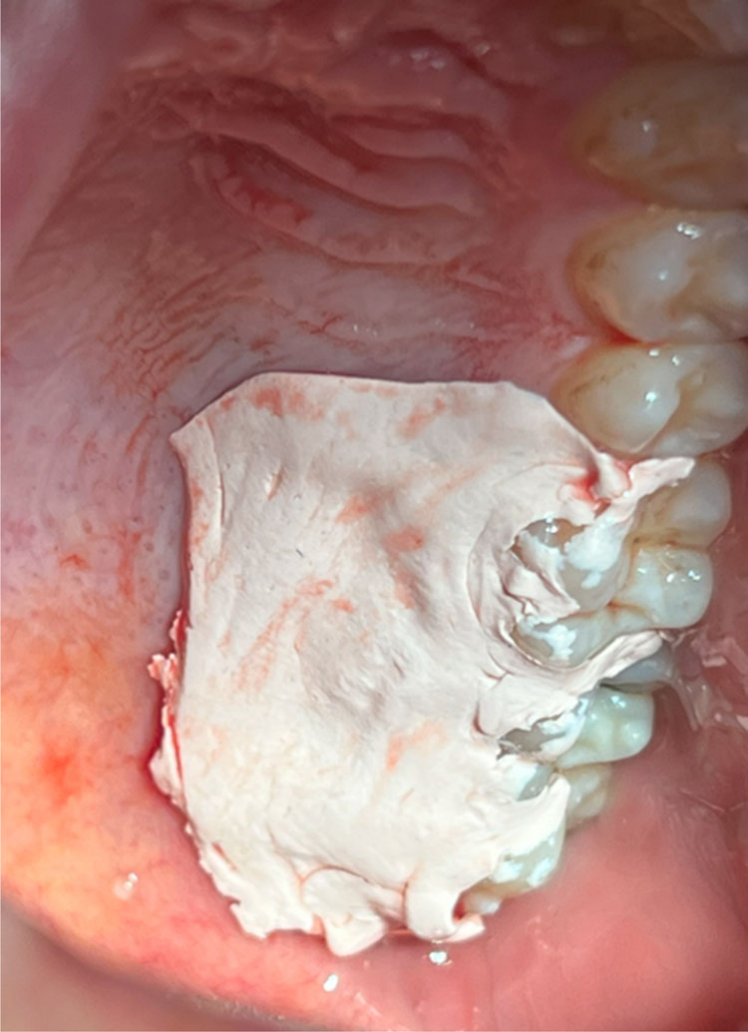
The application of Coe‐Pak dressing to the palatal donor site.

After harvesting the FGG, patients were randomly allocated to one of two study groups using online randomization software (random.org). In the control group, patients received conventional dressing, where the donor site was covered with Coe‐Pak (Coe Laboratories, Alsip, IL, USA) (Figure 2). The Coe‐Pack periodontal dressing was applied to the donor site to protect the healing tissues. The material was mixed and molded to fit the surgical site, ensuring a secure fit. Patients were instructed to avoid disturbing the dressing and to follow a soft diet to minimize the risk of detachment. Regular follow‐up appointments were scheduled to monitor the dressing's condition. In cases where detachment occurred, the dressing was promptly reapplied to maintain protection and support the healing process (Kathariya, Jain, and Jadhav 2015).

**Figure 3:**
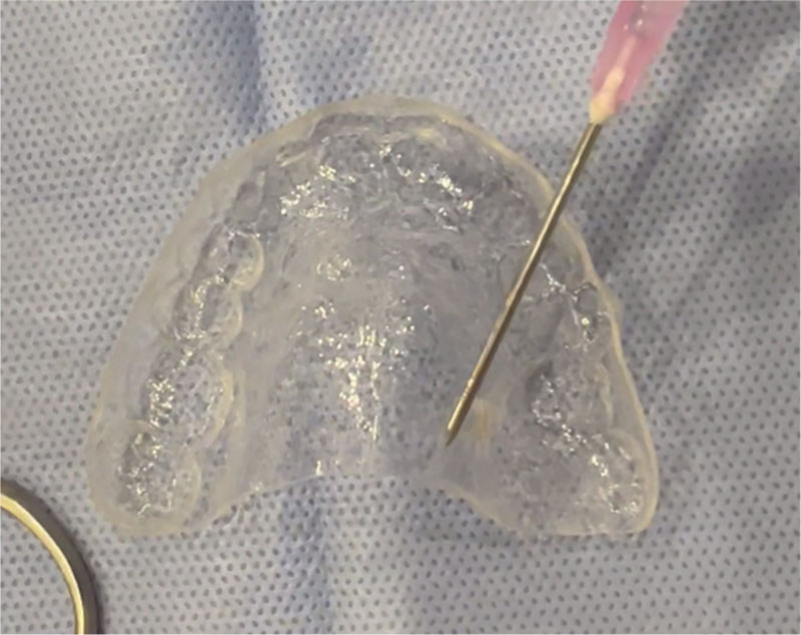
The application of PVP‐SH gel to the inside of the stent.

In contrast, patients in the test group were provided with a plastic stent made from a plastic shell of 1.5‐mm thickness on the patient's cast. Patients were instructed to apply 15 mL of PVP‐SH gel (Gelclair, Helsinn Healthcare SA, Switzerland) to the inside of the stent three times a day for 1 week, starting immediately after the surgical procedure (Figure 3). This protocol of administration was in accordance with the protocol provided by Hita‐Iglesias, Torres‐Lagares, and Gutiérrez‐Pérez (2006). Sufficient gel was supplied to the patient before leaving the clinic. Bleeding was controlled by applying pressure with a sterilized gauze before the patient's departure. We recognize that patients might not apply the treatment consistently, which could impact the study's outcomes. To mitigate this issue, we provided detailed instructions and conducted a training session for all patients on the proper application of the gel. Additionally, we scheduled regular follow‐up appointments to monitor adherence and address any concerns.

Each patient was administered 500 mg of amoxicillin three times daily (TID) for 8 days. Additionally, we advised the patients to rinse with 0.12% chlorhexidine gluconate (CHX) mouthwash twice daily for 3 weeks post‐procedure. Following the surgical intervention, patients were prescribed 500 mg of paracetamol for use if needed. We recommended that patients refrain from brushing the palatal aspect of their upper teeth until the follow‐up appointment, which was scheduled for 2 weeks post‐operation.

### 2.4 Post‐Surgical Assessment

The primary outcome, CE, was assessed every week for 1 month across both study groups, employing a 1% toluidine blue stain. The degree of staining at a site is indicative of its epithelialization status. Upon application of this stain, regions displaying a deep blue coloration signify the absence of epithelial tissue, as the dye has an affinity for connective tissue components. Conversely, areas presenting with a moderate blue hue suggest greater epithelialization. Sites devoid of any staining are indicative of full epithelialization (Dahiya et al. 2019). The epithelialization status was quantified using a scale where 0 indicates no epithelialization (deep blue), 1 indicates partial epithelialization (moderate blue), and 2 indicates CE (no staining). This scale allowed for a standardized assessment of the healing process at the donor site.

Additionally, secondary outcomes were assessed, such as Landry's healing index (Table 1; Landry 1985), which was documented every week for the same duration. The Landry healing scale was chosen for its simplicity, reliability, and widespread use in evaluating wound healing in periodontal and oral surgery studies. This scale provides a comprehensive assessment of tissue color, bleeding response, granulation tissue, and epithelialization, making it particularly relevant for monitoring the healing process post‐FGG harvesting (Ebrahimi et al. 2021; Kalaiyazhagi et al. 2023). Furthermore, the level of post‐operative pain was measured using a visual analog scale (VAS). During the initial week post‐surgery, patients rated their pain daily on the VAS, which ranged from 0 (no pain) to 100 (the most intense pain experienced by the individual). The number of analgesics consumed by a patient was documented daily for 1 week after the surgical intervention. Furthermore, at the study's conclusion, patients were queried regarding their willingness to undergo another FGG procedure, using a scale from 0 (absolutely unwilling) to 10 (I do not mind).

**Table 1:** Healing index by Landry et al.

Healing score	Explanation
1. Very poor	Tissue color: ≥ 50% of the gingiva red.
	Response to palpation: bleeding.
	Granulation tissue: present.
	Incision margin: not epithelialized, with loss of epithelium beyond the incision margin.
	Suppuration present.
2. Poor	Tissue color: ≥ 50% of the gingiva red.
	Response to palpation: bleeding.
	Granulation tissue: present.
	Incision margin: not epithelialized, with loss of epithelium beyond the incision margin.
3. Good	Tissue color: ≥ 25% and < 50% of the gingiva red.
	Granulation tissue: none.
	Incision margin: no connective tissue is exposed.
4. Very good	Tissue color: < 25% of the gingiva red.
	Response to palpation: no bleeding.
	Granulation tissue: none.
	Incision margin: no connective tissue is exposed.
5. Excellent	Tissue color: all the tissues are pink.
	Response to palpation: no bleeding.
	Granulation tissue: none.
	Incision margin: no connective tissue is exposed.

### 2.5 Statistical Analysis

The statistical analysis was performed using SPSS (version 29.0.2.0; IBM Corp., Armonk, NY, USA). The categorical variables were expressed as percentages, and the quantitative data were expressed as mean ± standard deviation. The distribution of data was assessed by the Kolmogorov–Smirnov test. The significance level was set at *p* < 0.05. The Mann–Whitney *U* test was performed to determine the differences between two independent groups, and for intergroup comparisons during follow‐up periods, the Wilcoxon signed‐rank test was used.

## 3 Results

**Figure 4:**
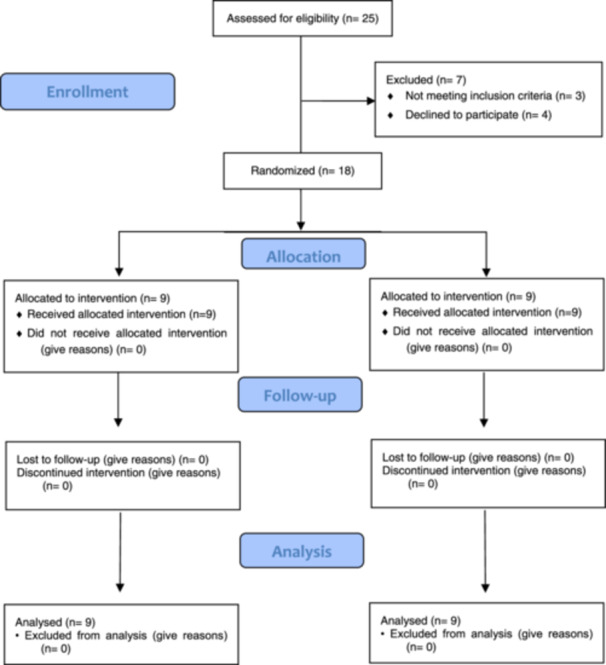
CONSORT flow diagram.

Twenty‐five patients were eligible for this study. However, seven patients were excluded: three did not meet the inclusion criteria, and four declined to participate (Figure 4). In total, 18 patients participated in the study and underwent FGG application in the lower anterior arch. This procedure was indicated for lack of keratinized tissue.

### 3.1 Demographic Data

The mean age of patients was 30.2 ± 4.1 years in the control group and 31.4 ± 2.9 years in the test group. These figures are compared to the mean age of the entire sample, which was 30.8 ± 3.5 years. The test group comprised more male participants (six males) compared to the control group (four males). However, no statistically significant differences were observed regarding the patients' ages or the gender distribution between the groups (*p* = 0.10 and *p* = 1.08), respectively. Demographic characteristics of the included patients are presented in Table 2.

**Table 2:** Demographic characteristics of the included participants in each group.

Groups	Age, mean ± SD	*p* value	Sex, *N* (%)		*p* value
			Men	Female	
Control group (*n* = 9)	30.2 ± 4.1	0.10	4 (44.44%)	5 (55.55%)	1.08
Test group (*n* = 9)	31.4 ± 2.9		6 (66.66%)	3 (33.33%)	

Abbreviation: SD, standard deviation.

### 3.2 Graft Dimensional Assessment

The average thickness of the FGGs measured 2.1 ± 0.3 mm in the test group and 2.3 ± 0.4 mm in the control group. Comparative analysis revealed no statistically significant differences in graft dimensions (thickness, length, and width) (*p* < 0.05). The specific graft measurements for each group are detailed in Table 3.

**Table 3:** Graft dimensions in two groups.

Graft dimensions (mm)	Control group	Test group	*p* value
	Mean ± SD	Mean ± SD	
Palatal thickness	4.22 ± 0.9	4.22 ± 1.2	0.178
Graft thickness	2.3 ± 0.4	2.1 ± 0.3	0.463
Graft length	15.8 ± 5.3	16.2 ± 4.9	0.329
Graft width	6.3 ± 0.9	6.7 ± 1.2	0.627

### 3.3 Compared Outcomes

Analgesic consumption was lower in the test group than in the control group. Statistically significant differences were noted only during the first 3 days post‐surgery (*p* < 0.05). The test group also reported lower scores on the VAS compared to the control group, with significant differences lasting for 4 days post‐surgery (*p* < 0.05), indicating a reduced pain index. An intragroup assessment showed a decreasing pain index over time in both groups. Table 4 details the analgesic usage and VAS scores. Interestingly, the willingness to undergo retreatment was significantly higher in the test group (*p* = 0.008), with mean agreement scores of 7.11 ± 4.14 in the test group and 1.67 ± 2.50 in the control group.

**Table 4:** Data of analgesics consumed and VAS between the groups.

Day of follow‐up	Analgesics consumed				Visual analog scale			
	Control	Test	*U* value for Mann–Whitney	*p* value	Control	Test	*U* value for Mann–Whitney	*p* value
1st day	6.94 ± 2.4	12.06 ± 3.1	17.5	0.018*	70.56 ± 16.48	51.11 ± 11.40	11.5	0.009
2nd day	6.89 ± 2.2	10.11 ± 2.7	17.0	0.020*	57.22 ± 20.02	35.56 ± 8.46	16.0	0.025
3rd day	6.78 ± 1.9	8.22 ± 2.5	16.0	0.017*	40.56 ± 16.48	25.00 ± 8.66	16.5	0.026
4th day	6.50 ± 1.6	6.64 ± 1.7	40.5	1.000	22.22 ± 10.34	12.22 ± 4.41	15.0	0.015
5th day	5.19 ± 1.0	5.19 ± 1.0	40.5	1.000	2.78 ± 5.65	0	31.5	0.146
6th day	3.03 ± 0.7	3.03 ± 0.7	40.5	1.000	0	0	40.5	1.000
7th day	1.7 ± 0.5	1.7 ± 0.5	40.5	1.000	0	0	40.5f	1.000

* Statistically significant difference.

Significant differences in wound healing and epithelization were observed between the groups (*p* < 0.05). The test group demonstrated superior wound healing compared to the control group during the first 3 weeks, with a mean rank of 11.50 for the test group and 7.50 for the control group. The rate of complete epithelization was also significantly higher in the test group during this period (*p* < 0.05). By the fourth week, both groups exhibited substantial epithelization, achieving 96%–100%, as shown in Table 5.

**Table 5:** Wound healing and complete wound epithelization between groups.

	Wound healing (mean rank)			Complete wound epithelization (%)		
	Control	Test	*p* value	Control	Test	*p* value
1st week	7.50 ± 0.61	11.50 ± 0.66	0.001*	13.1	44.4	0.028*
2nd week	7.50 ± 0.61	11.50 ± 0.66	0.001*	47.8	55.6	0.028*
3rd week	7.50 ± 0.61	11.50 ± 0.66	0.001*	84.3	100	0.028*
4th week	7.00 ± 0.66	12.00 ± 0.51	0.068	95.9	100	0.065

* Statistically significant difference.

No complications were encountered during the study. All patients tolerated the procedures well, and there were no adverse events or unexpected outcomes reported in either the control or test groups. This indicates that both Coe‐Pak and PVP‐SH gel are safe and well‐tolerated dressing materials for managing the donor site post‐FGG harvesting.

## 4 Discussion

In this study, our findings demonstrated that PVP‐SH gel significantly improves clinical outcomes and patient satisfaction following FGG harvesting compared to Coe‐Pak. Patients treated with PVP‐SH gel experienced less pain, required fewer analgesics, and reported a higher willingness to undergo similar treatments in the future. These findings suggest that PVP‐SH gel not only enhances the healing process at the donor site but also positively impacts the overall patient experience. Reduced pain severity and lower analgesic consumption are particularly noteworthy, as they align with the current focus on minimizing post‐operative discomfort and improving the quality of care in dental surgeries (Rawal 2016).

The off‐label use of PVP‐SH gel in the oral cavity, specifically as a dressing material for FGG donor sites, warrants careful consideration of its safety and usability. Although PVP‐SH gel was not originally marketed for this specific application, its components have well‐documented safety profiles and are used in various medical and dental contexts.

PVP is a hydrophilic polymer widely used in pharmaceuticals as a binder and film‐forming agent. It is known for its chemical and biological neutrality, making it safe for use in the oral cavity. PVP is also used in oral and topical formulations, indicating its compatibility with mucosal tissues. Sodium hyaluronate is a naturally occurring polysaccharide found in various body tissues, including the skin and joints. It is commonly used in medical and dental products for its viscoelastic and mucoadhesive properties, which help in tissue hydration and protection. Sodium hyaluronate has been extensively studied and is considered safe for use in the oral cavity, as evidenced by its inclusion in products for treating OM and other inflammatory conditions (Buchsel 2008).

Numerous research efforts have highlighted the beneficial impact of PVP‐SH gel in treating various inflammatory conditions. Rezazadeh et al. developed and tested an alternative formulation inspired by PVP‐SH gel with certain modifications. Their study on rats with artificially induced OM revealed that this new formulation effectively mitigated the condition's severity, performing on par with the established PVP‐SH gel product (Rezazadeh et al. 2021). In a separate investigation, Barber et al. evaluated PVP‐SH gel's role in alleviating pain for mucositis patients undergoing intensive therapy for head and neck cancer. Their findings suggest that PVP‐SH gel matches the effectiveness of the standard treatment for managing pain resulting from radiotherapy‐induced OM (Barber et al. 2007). In research conducted by Berndtson, a cohort of 10 head and neck cancer patients undergoing radiotherapy was examined (Berndtson 2001), whereas De Cordi et al. analyzed the experiences of 30 patients undergoing chemotherapy for cancers at various sites, including the breast, colorectum, lungs, stomach, and head and neck (De Cordi et al. 2001). A separate investigation by Innocenti and associates focused on 33 individuals with intraoral lesions, with causes ranging from AIDS to undetermined origins (Innocenti, Moscatelli, and Lopez 2002). This study found that 57% of participants experienced a significant reduction in pain lasting over 3 h, and 40% reported pain relief for 2–3 h. Additionally, there was an enhancement in the patients' capacity to consume food and liquids from the initial assessment to the follow‐up period of 7–10 days. However, the absence of OM grading or details on concurrent treatments leaves room for the possibility that the observed symptom improvements could be attributed to the mouth's natural healing processes. The findings reported by De Cordi et al. echoed these results, showing notable alleviation of pain, OM severity, and swallowing difficulties. Yet, the lack of information on ongoing cancer treatments or other potential influencing factors suggests that general oral health improvements might account for the observed benefits. Berndtson's research presents challenges in evaluation due to the unspecified methodology and the fact that all subjects were receiving various types of pain management, including potent opioids.

Although the use of PVP‐SH gel for FGG donor sites is off‐label, the individual components' established safety profiles and the positive outcomes observed in our study suggest that this application is both safe and beneficial. However, further studies with larger sample sizes and long‐term follow‐up are recommended to confirm these findings and support regulatory approval for this specific use.

Sodium hyaluronate, a derivative of hyaluronic acid (HA) and one of the primary components of PVP‐SH gel, has been extensively studied for its beneficial effects on wound healing and pain management in various clinical settings. Recent studies have shown that the topical application of HA in conjunction with FGG can significantly reduce post‐operative pain and improve wound healing at the donor site (Khalil et al. 2022; Silva, de Souza, and Nogueira 2023). For instance, an RCT demonstrated that HA application on both donor and recipient sites resulted in lower pain scores and improved patient satisfaction compared to the control group (Khalil et al. 2022). Another study highlighted the effectiveness of HA in reducing post‐operative morbidity and accelerating healing in palatal donor sites (Silva, de Souza, and Nogueira 2023). This scoping review identified various interventions, including HA, that showed potential for improving patient‐reported outcomes and reducing post‐operative discomfort and pain (Silva, de Souza, and Nogueira 2023). It also emphasized the importance of HA in promoting wound healing and reducing pain at the donor site. Additionally, a study evaluating the effect of topical HA application on FGG donor and recipient sites during the early wound healing period showed positive effects on wound healing (Çankaya et al. 2020). Furthermore, a study comparing the effects of topical HA, hypochlorous acid, and flurbiprofen on post‐operative morbidity of palatal donor sites after FGG surgery demonstrated the effectiveness of HA in reducing pain and accelerating healing (Alpan and Cin 2023). These findings support the use of PVP‐SH gel as a dressing material in FGG procedures, as the HA component contributes to its overall efficacy. The significant preference for PVP‐SH gel among patients indicates its potential as a preferred dressing material in periodontal procedures, warranting its consideration for broader clinical application.

Given the demonstrated efficacy of PVP‐SH gel in managing various inflammatory conditions and oral lesions, it is important to consider its use in managing FGG donor sites. The positive outcomes observed in other clinical settings suggest that PVP‐SH gel could offer similar benefits in periodontal procedures, enhancing the healing process and improving patient comfort. The significant preference for PVP‐SH gel among patients further supports its potential as a preferred dressing material in periodontal procedures, warranting its consideration for broader clinical application.

However, our study is not without limitations. Although the sample size was sufficient for initial conclusions, it invites further research with a larger cohort to confirm these findings. Future studies should also consider the long‐term effects of PVP‐SH gel and its potential for integration into routine clinical practice.

## 5 Conclusion

Our study indicates that PVP‐SH gel significantly outperforms Coe‐Pak as a dressing material post‐FGG procedures. Patients treated with the gel reported less pain, required fewer analgesics, and showed faster wound healing and epithelialization. No complications were encountered, confirming the safety and tolerability of both dressings. These findings suggest that PVP‐SH gel could improve post‐operative recovery and patient satisfaction, advocating for its broader clinical application in periodontal therapies.

## Author Contributions

Mohammad Baroudi conceived the research idea and performed the treatment. Mohammad Baroudi and Ali Omair interpreted the data and drafted, revised, formatted, and edited the manuscript. Majd Othman co‐conceived the research idea and supervised Mohammad Baroudi's master's thesis. All authors gave their approval to the final version of this manuscript.

## Ethics Statement

The Institutional Ethics Committee of Damascus University approved this study (approval number UDDS‐28066141/SRC‐2215).

## Conflicts of Interest

The authors declare no conflicts of interest.

## Data Availability

The data that support the findings of this study are available from the corresponding author upon reasonable request. Pseudonymized data can be obtained upon reasonable requisition from the corresponding author.
